# How to manage multiple food allergies in children

**DOI:** 10.1186/2045-7022-1-S1-S26

**Published:** 2011-08-12

**Authors:** Susanne Lau

**Affiliations:** 1 Charité, Berlin, Germany

## 

Approximately 25% of infants with atopic dermatitis are found to be affected by food allergy. In Germany, overall prevalence of food allergy in infants and preschool children is 1-3%. Although several studies have examined the prevalence of food allergy, for instance longitudinally in the EuroPrevall study, there is less information on multiple food allergy. Estimates of prevalence of children allergic to multiple foods is difficult to ascertain because those with allergy to one food may avoid additional foods for concerns related to cross-reactivity, positive tests, or suspected reactions, or they may be reluctant to introduce foods known to be common allergens. Diagnosis relies on accurate history, skin or serum-IgE testing and supervised food challenge. Reasonable diets, patient education and emergency medications can help to manage multiple food allergies. However, there is a considerable burden on caregivers in terms of social limitations, and impaired quality of life due to various reasons. Especially caregivers, whose children had been to the emergency department for food allergy, experience a negative impact on their life.

